# Influenza Vaccination and Risk of Lung Cancer in Patients with Chronic Kidney Disease: A Nationwide, Population-Based Cohort Study

**DOI:** 10.3390/cancers14122926

**Published:** 2022-06-14

**Authors:** Chun-Chao Chen, Chia-Hsien Wu, Cheng-Hsin Lin, Chun-Chih Chiu, Tsung-Yeh Yang, Meng-Huan Lei, Hsien-Tang Yeh, William Jian, Yu-Ann Fang, Wen-Rui Hao, Ju-Chi Liu

**Affiliations:** 1 Division of Cardiology, Department of Internal Medicine, Shuang Ho Hospital, Taipei Medical University, New Taipei City 235, Taiwan; b101092035@tmu.edu.tw (C.-C.C.); b101103047@tmu.edu.tw (C.-H.W.); 17257@s.tmu.edu.tw (C.-C.C.); 15535@s.tmu.edu.tw (T.-Y.Y.); runawayyu@hotmail.com (Y.-A.F.); 2 Taipei Heart Institute, Taipei Medical University, Taipei 110, Taiwan; chlin@s.tmu.edu.tw; 3 Division of Cardiology, Department of Internal Medicine, School of Medicine, College of Medicine, Taipei Medical University, Taipei 110, Taiwan; 4 Graduate Institute of Medical Sciences, College of Medicine, Taipei Medical University, Taipei 110, Taiwan; 5 Department of Medical Education, National Taiwan University Hospital, Taipei 100, Taiwan; 6 Division of Cardiovascular Surgery, Department of Surgery, Shuang Ho Hospital, Taipei Medical University, New Taipei City 235, Taiwan; 7 Division of Cardiovascular Surgery, Department of Surgery, School of Medicine, College of Medicine, Taipei Medical University, Taipei 110, Taiwan; 8 Cardiovascular Center, Lo-Hsu Medical Foundation Luodong Poh-Ai Hospital, Yilan 265, Taiwan; mhlei6401@yahoo.com.tw; 9 Department of Surgery, Lotung Poh-Ai Hospital, Luodong 265, Taiwan; 836023@mail.pohai.org.tw; 10 Department of Emergency Medicine, University Hospitals Cleveland Medical Center, Cleveland, OH 44106, USA; william.jian@gmail.com; 11 Graduate Institute of Clinical Medicine, College of Medicine, Taipei Medical University, Taipei 110, Taiwan

**Keywords:** chronic kidney disease, influenza vaccination, lung cancer

## Abstract

**Simple Summary:**

The risk of lung cancer occurrence among patients with chronic kidney disease (CKD) is significantly higher than patients with normal renal function. Moreover, the long-term outcomes of CKD patients with lung cancer are poor. We conducted the present nationwide population based study to assess the association between the risk of lung cancer and influenza vaccination. The results showed that among CKD patients, potentially risk reduction of lung cancer was observed after influenza vaccination. In addition, the potentially protective effect of influenza vaccination on lung cancer risk reduction appeared to be dose dependent. The findings of present study provided the additional effect of influenza vaccination among patients with CKD.

**Abstract:**

Chronic kidney disease (CKD) is significantly associated with lung cancer incidence. The aim of this study was to elucidate whether influenza vaccination reduces the incidence of lung cancer in patients with CKD. This cohort study enrolled patients with a record of CKD diagnosis from 2000 to 2012 in Taiwan’s National Health Insurance Research Database. Included patients were divided into vaccinated and unvaccinated groups. In total 12,985 patients with CKD were enrolled. Among these patients, 5495 were vaccinated and 7490 were unvaccinated. The risk of lung cancer was significantly lower in the influenza vaccination group after adjusting for age, sex, dialysis status, lung diseases, comorbidities, level of urbanization, and monthly income (adjusted hazard ratio (HR): 0.50, 95% confidence interval (CI; 0.38–0.65), *p* < 0.05). Lower risk of lung cancer was observed in both sexes, all age groups, dialysis status and co-existed lung diseases. The association between the risk of lung cancer and vaccination appeared to be dose-dependent (adjusted HRs: 0.91 (0.66–1.25), 0.49 (0.34–0.71), and 0.25 (0.17–0.38) for patients who received 1, 2 or 3, and ≥4 vaccinations during the follow-up period, respectively). In conclusion, Influenza vaccination decreased the risk of lung cancer in patients diagnosed with CKD. This potentially protective effect against lung cancer appeared to be dose dependent.

## 1. Introduction

Chronic kidney disease (CKD) originates through various pathophysiological pathways and destroys the structure and function of the kidney over months or years. Epidemiological studies have determined the prevalence of CKD to be approximately 11% in high-income countries, although prevalence varies widely [1]. Although CKD is most commonly associated with higher cardiovascular mortality, cancer-related mortality has also been reported as being higher in CKD by 20% (adjusted hazard ratio (HR): 1.2, 95% confidence interval (CI; 1.02–1.42)) [2]. Furthermore, reports have revealed that patients with CKD, both those undergoing renal replacement therapy (RRT) and those not, have an increased risk of cancer. However, population-based cohort studies have indicated that patients on dialysis have a higher risk of cancer compared with patients with CKD who have not yet undergone RRT (27–50% and 8–25% increased risks, respectively) [3,4,5]. The estimated glomerular filtration rate (eGFR) is closely associated with the higher incidence and mortality of cancer in patients with CKD or undergoing dialysis. For every 10 mL/min/1.73 m^2^ decrement in the eGFR, the risk of cancer increases by 29% (adjusted HR: 1.29, 95% CI 1.10–1.53) and that of cancer-related mortality increases by 18% [6,7]. Therefore, patients with CKD have an increased risk of cancer and cancer-related mortality. Furthermore, several studies have demonstrated that CKD was significantly associated with the incidence of lung cancer [4,6,8].

Chronic inflammation, which is an essential feature of CKD, is the key pathogenesis of tumor growth and malignant transformation [9,10,11]. Patients with CKD experience not only an exaggerated reaction of the immune system but also impairment of their innate and adaptive immune responses [12]. Furthermore, the risk of hospitalization with infection increases as the eGFR decreases; compared with an eGFR ≥60 mL/min/1.73 m^2^, the risk increases 2–3 times with an eGFR <30 mL/min/1.73 m^2^ [13]. Chronic inflammation resulting from viral infection significantly increases the likelihood of cancer development. In addition, the presence of oncoviruses may induce an oncogenic effect by activating inflammatory pathways and cytokines [14]. Previous animal studies have revealed that the influenza virus can lead to tumorigenesis, and a general population cohort study in Taiwan demonstrated that patients who were exposed to the influenza virus had a 9% higher risk of lung cancer (aOR: 1.09, 95% CI 1.04–1.14, *p* < 0.0001) [15,16,17].

Because the influenza vaccine may be the most cost-effective strategy for preventing influenza-related diseases and because lung cancer has been found to more commonly occur in patients with CKD due to their chronic inflammation and increased inflammatory mediators—possibly the result of repeated or chronic virus infections, we investigated whether administering available influenza vaccinations to such patients would reduce the incidence of lung cancer among them. We performed a population-based cohort study by using reimbursement claims collected from Taiwan’s National Health Insurance Research Database (NHIRD) to illuminate the potential protective benefits of an influenza vaccination against cancer in Taiwanese patients with CKD.

## 2. Methods

The National Health Insurance (NHI) program in Taiwan has been in effect since 1995; the NHI program provides comprehensive health care for Taiwan residents of all ages, and approximately 98% of the over 23 million residents of Taiwan are currently enrolled in the program. The NHIRD, established and administered by the National Health Insurance Administration, contains the claims data of NHI enrollees; we analyzed relevant data from 2000 to 2012. No statistically significant differences in sex, age, or health care costs were noted between the collected sample and all enrollees [18]. Data in the NHIRD are deidentified to ensure anonymity and prevent the identification of specific persons/institutions, including patients, medical institutions, or physicians, involved in the provision of health care; this delinking of relationships occurs before data are sent to the National Health Research Institutes for database construction, and further deidentification is achieved before the data are released to researchers. Furthermore, all researchers using the NHIRD and its data subsets must sign a written agreement declaring that they have no intention to obtain information that would infringe upon the privacy of patients or care providers [19]. 

**Figure 1:**
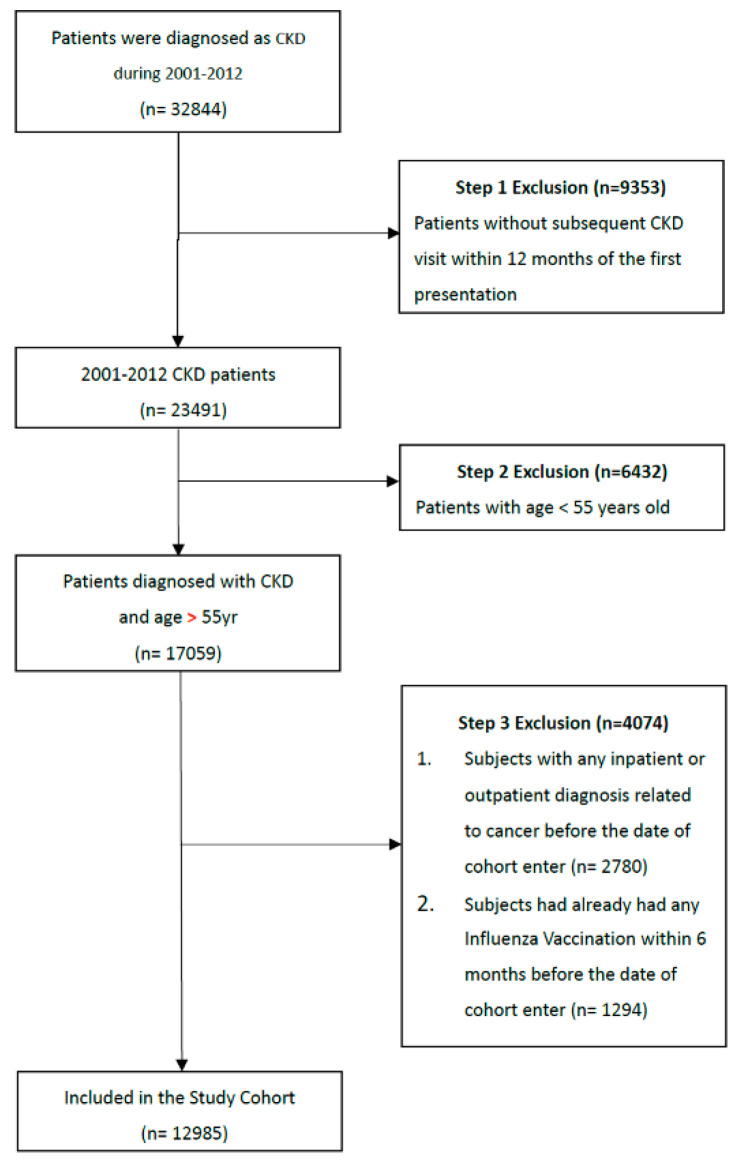
Data selection process. A total of 32,844 patients with CKD diagnoses were enrolled during 2001 to 2012. Patients diagnosed with CKD without subsequent outpatient or inpatient visits, aged less than 55 years, or with pre-existing cancer or a history of influenza vaccination were excluded. A final total of 12,985 patients were enrolled in the study.

Our study cohort comprised all patients diagnosed with CKD (according to the International Classification of Diseases, Ninth Revision, Clinical Modification [ICD-9-CM] code 585) who visited health care facilities in Taiwan (n = 32,844) from 1 January 2001, to 31 December 2012. Patients without a subsequent outpatient visit, emergency department visit, or inpatient hospitalization for CKD within 12 months of the first presentation were considered as not having CKD and were, therefore, excluded (n = 9353; Figure 1). Moreover, we used a 1-year (2000) washout period to ensure that no patients in this cohort had cancer before enrollment. Individuals who were younger than 55 years (n = 6432) or who had a history of any inpatient or outpatient diagnosis related to cancer before the enrollment date (n = 2780) were excluded. Patients vaccinated within 6 months before diagnosis with CKD were also excluded (n = 1294). Our final study cohort comprised 12,985 patients who had received a diagnosis of CKD in Taiwan over the 12-year study period. Of these, 5495 and 7490 patients did and did not receive influenza vaccination, respectively.

In Taiwan, influenza vaccination has been free of charge and recommended for high-risk adults aged ≥50 years (i.e., those with type 2 diabetes, chronic liver infection or cirrhosis, cardiovascular diseases, or chronic pulmonary diseases) since 1998 [20]. Vaccination status was identified using the ICD-9-CM code V048 or drug codes that confirmed receipt of the vaccine. Each patient included in the study had been followed up to assess the presence of risks of lung cancer or of protective factors. These included age; sex; Charlson comorbidity index score (CCI); dialysis status; presence of lung diseases (chronic obstructive pulmonary disease [COPD], lower respiratory tract infection, or influenza); presence of comorbidities (diabetes, hypertension, or dyslipidemia); use of a statin, metformin, aspirin, or other drug that acts on the renin–angiotensin–aldosterone system (RAA; including angiotensin-converting enzyme inhibitors (ACEIs), angiotensin II receptor blockers (ARBs), and aldosterone-receptor antagonists (ARAs)); urbanization level; and monthly income [21,22]. The endpoint was the occurrence of lung cancer (ICD-9-CM code 162.X) with a subsequent outpatient visit, emergency department visit, or inpatient hospitalization for lung cancer within 12 months; death; withdrawal from the NHI program; or 31 December 2012. Unvaccinated patients served as a reference arm.

## 3. Statistical Analysis

Propensity scores (PSs) were used to reduce selection bias and estimate the effects of vaccination by accounting for the covariates predictive of receiving the intervention (vaccine) with a logistic regression model [23]. The covariates in the main model were adjusted for PSs for age, sex, CCI, dialysis status, lung diseases, comorbidities, monthly income (0, NT$1 to NT$21,000, NT$21,000 to NT$33,300, and >NT$33,300), and level of urbanization (urban, suburban, and rural; Table 1). Chi-squared analyses were used to identify differences between the vaccinated and unvaccinated groups with respect to dialysis status, lung diseases, comorbidities, demographic variables, and socioeconomic status. The HR and 95% CI for the association between influenza vaccination and the incidence rate of lung cancer were examined using a Cox proportional hazards regression analysis. A stratified analysis was conducted to evaluate the effects of vaccination in different age group, sex, dialysis status, or lung diseases (Table 2). The dose-dependent effects of influenza vaccination on the incidence of lung cancer were also examined in four categories of patients: unvaccinated and 1, 2 or 3, and more than 4 total vaccinations. When conducting epidemiologic database studies, in sensitivity analyses, external adjustments can improve the understanding of the effects of drugs and other covariates on outcomes. Hence, in the sensitivity analysis for this study, adjustments were made to estimate the association of age and sex; CCI score; dialysis; COPD, lower respiratory tract infection, influenza, diabetes, hypertension, and dyslipidemia; and the use of a statin, metformin, RAA, and aspirin with the incidence of lung cancer in various models. The models were adjusted for covariates in the main model and each additional covariate (Table 3). All statistical analyses were conducted using Statistical Package for the Social Sciences (SPSS) software package Version 22.0 (SPSS Inc, Chicago, IL, USA) and SAS 9.4 software (SAS Institute Inc., Cary, NC, USA). The significance criterion was set at *p* < 0.05.

## 4. Results

### 4.1. Baseline Characteristics among Vaccinated and Unvaccinated Groups

The eligible study population of the cohort consisted of 12,985 individuals, with 5495 influenza-vaccinated and 7490 unvaccinated patients, accounting for 42.3% and 57.7% of the study population, respectively. Significant differences were observed between the vaccinated and unvaccinated groups in the distributions of age, socioeconomic status, and urbanization level. The unvaccinated group also exhibited a higher prevalence of lung diseases and underlying comorbidities, including high CCI scores, lower respiratory tract infection, influenza, diabetes, hypertension, and dyslipidemia, before PS adjustment. A higher percentage of patients in the vaccinated group were under dialysis. In addition, patients in the vaccinated group had taken drugs for chronic diseases for longer periods (Table 1). The mean period of time for follow-up in vaccinated group was 6.19 person-years, and the mean period of time for follow-up in unvaccinated group was 2.93 person-years (Table 2).

### 4.2. Sex and Age among Vaccinated and Unvaccinated Groups

The incidence rate of lung cancer was significantly lower in the influenza vaccination group, with an adjusted HR of 0.50 (95% CI 0.38–0.65; *p* < 0.05) when compared with the unvaccinated group (Table 2). Similar protective effects were also found in both sexes and in all older adult groups. The adjusted HR was 0.39 (95% CI 0.21–0.70; *p* < 0.05), 0.50 (95% CI 0.33–0.76; *p* < 0.05), and 0.49 (95% CI 0.32–0.72; *p* < 0.05) in the subgroups of participants aged 55–64, 65–74, and over 75 years. The adjusted HR were 0.39 (95% CI 0.24–0.65; *p* < 0.05) in women and 0.55 (95% CI 0.40–0.74; *p* < 0.05) in men, when compared with their unvaccinated counterparts. Furthermore, we observed age and sex to have effects on the incidence rate of lung cancer. The incidence rate of lung cancer was positively related to age; the incidence rate was 426.4 (95% CI 303.2–549.6), 652.1 (95% CI 450.0–854.2), and 1080.5 (95% CI 792.3–1368.7) per 10^5^ person-years in the unvaccinated group, whereas incidence was 166.7 (95% CI 82.3–251.1), 347.6 (95% CI 255.7–439.5), and 534.3 (95% CI 384.7–683.9) per 10^5^ person-years in the vaccinated group at the ages of 55–64, 65–74, and 75 years and older, respectively. Men had a higher incidence rate than women, with an incidence rate of 781.6 (95% CI 625.2–937.9) and 483.9 (95% CI 385.1–582.8) per 10^5^ per-son-years for unvaccinated and vaccinated men, respectively, and 456.6 (95% CI 321.7–591.5) and 180.2 (95% CI 112.3–248.2) per 10^5^ person-years for unvaccinated and vaccinated women, respectively (Table 2).

### 4.3. Dialysis and Lung Diseases among Vaccinated and Unvaccinated Groups

The adjusted HRs were 0.55 (95% CI 0.35–0.88; *p* < 0.05) and 0.47 (95% CI 0.34–0.64; *p* < 0.05) in vaccinated patients under dialysis and patients without dialysis, respectively, compared to unvaccinated ones. Among patients with and without history of COPD, the adjusted HR were 0.44 (95% CI 0.30–0.64; *p* < 0.05) and 0.52 (95% CI 0.36–0.76; *p* < 0.05) respectively. The adjusted HR in patients with lower respiratory tract infection was 0.51 (95% CI 0.31–0.85; *p* < 0.05) and in patients without lower respiratory tract infection was 0.47 (95% CI 0.34–0.63; *p* < 0.05), respectively. The adjusted HR was 0.50 (95% CI 0.30–0.82; *p* < 0.05) and 0.50 (95% CI 0.37–0.67; *p* < 0.05) in the subgroup of influenza infection and without influenza infection (Table 2). 

### 4.4. Sensitivity Analysis

In the sensitivity analysis, adjustments were made to examine the association of various covariates (e.g., comorbidities, demographic variables, and socioeconomic status) with the incidence of lung cancer in different models (Table 3). Influenza vaccination significantly reduced the incidence rate among the main model and subgroups of various covariates. The analysis further demonstrated a dose-dependent protective effect, where influenza-vaccinated patients had a lower adjusted HR with an increased total number of vaccinations in the main model and subgroup analysis. Moreover, the dose effect was stronger when patients received more than two doses of influenza vaccination. The adjusted HRs for lung cancer risk were 0.91 (95% CI 0.66–1.25), 0.49 (95% CI 0.34–0.71; *p* < 0.05), and 0.25 (95% CI 0.17–0.38; *p* < 0.05) among patients who received 1, 2 or 3, and ≥4 vaccinations during the follow-up period, respectively. Furthermore, the protective effects of influenza vaccination were unclear if patients received only a single dose of the vaccine during the study period. We found no significant differences between the unvaccinated and one-dose-vaccinated groups in either the main model or any subgroup analysis. In addition, patients with younger age required more vaccinations to acquire protective effects against lung cancer. No significant differences were evident until patients aged 55–64 received ≥4 vaccinations. The adjusted HR in the subgroups of patients aged 55–64 was 0.56 (95% CI 0.25–1.24), 0.49 (95% CI 0.21–1.15), and 0.14 (95% CI 0.03–0.58; *p* < 0.05) in the 1, 2 or 3, and ≥4 vaccination groups, respectively. Moreover, patients under dialysis should receive ≥4 vaccinations to ensure the protective effect of influenza vaccine. The adjusted HR in the subgroups of patients under dialysis was 0.84 (95% CI 0.46–1.54), 0.65 (95% CI 0.36–1.17), and 0.27 (95% CI 0.12–0.58; *p* < 0.05) in the 1, 2 or 3, and ≥4 vaccination groups, compared to unvaccinated group. The adjusted HR in the subgroup of patients without dialysis was 0.93 (95% CI 0.63–1.36), 0.42 (95% CI 0.26–0.66; *p* < 0.05), and 0.24 (95% CI 0.15–0.39; *p* < 0.05) in the 1, 2 or 3, and ≥4 vaccination groups (Table 3). 

## 5. Discussion

In this population-based cohort study, we found that patients with CKD vaccinated against influenza had an approximately 50% lower risk of lung cancer. Vaccination as a means of preventing cancer has been demonstrated and is recommended for cancers associated with viral infection; for example, the hepatitis B vaccine is recommended to prevent hepatocellular carcinoma, and the human papillomavirus (HPV) vaccine is recommended for HPV-related cervical and other cancers [5,24]. In a previous study, the association between influenza vaccination in patients with chronic obstructive pulmonary disease and risk of lung cancer was investigated, and the risk of lung cancer was revealed to be significantly reduced after influenza vaccination (adjusted HR = 0.40, 95% CI 0.35–0.45; *p* < 0.001) [18]. The present study is the first population-based cohort study to demonstrate that influenza vaccination may reduce the incidence of lung cancer in patients with CKD.

Two possible mechanisms may explain this phenomenon. The first is chronic inflammation [25]. In addition to the decrement of renal function, numerous uremic toxins accumulate in patients with CKD. Middle molecules (<58 kDa), including many cytokines and other proinflammatory mediators that can cross the glomerular filtration barrier under normal conditions, cannot be removed through dialysis [9,26]. A prospective observational cohort study revealed plasma cytokine levels to be significantly higher among participants with lower levels of eGFR, with 2-fold, 2.1-fold, and 41% higher levels of interleukin (IL)-6, tumor necrosis factor alpha (TNF-α), and IL-1 receptor antagonist, respectively, in patients with eGFR <30 mL/min per 1.73 m^2^ compared with those with eGFR >60 mL/min per 1.73 m^2^ [27]. Patients with CKD are characterized by an imbalance between pro-and anti-inflammatory cytokines, which are associated with poor disease outcomes [28]. Proinflammatory cytokines, such as IL-6, IL-1β, and TNF-α, have been well recognized as promoting inflammation-associated carcinogenesis. The activation of the glycoprotein 130 receptor by IL-6 can activate several oncogenic signaling cascades, including Ras/ERK, JAK/STAT3, and PI3K/Akt. Furthermore, IL-1β regulates the expression of several proteins, such as the transcription factor NF-κB. These proinflammatory transcription factors are critical mediators of inflammation-related cancer [11]. Patients with CKD, who have impaired immune systems, tend to develop pneumonia or even sepsis when infected by the influenza virus [12,28,29]. Influenza infection–induced inflammation reinforces the cytokines cascade, which patients with CKD have difficulty overcoming and which can promote cancer development [30]. This mechanism may explain why patients with a lower eGFR have a higher incidence rate of cancer [6]. The influenza vaccine may be the most cost-effective method for preventing such an outcome. In the cohort study, both of CKD patients under dialysis and patients did not receive dialysis had significant lower risk of lung cancer after influenza vaccination. However, patients under dialysis should receive more vaccinations to ensure the protective effect. These findings reinforced the above theories.

The second possible mechanism involves reactive oxygen species (ROS), which may play an essential role in lung cancer developing in patients with CKD [31]. Increased intracellular levels of ROS can lead to oxidation of lipids, DNA, and proteins, contributing to cellular damage. By contrast, antioxidant systems, such as superoxide dismutase, catalase, or glutathione peroxidase, maintain a relatively low ROS level, which can result in cell proliferation and growth [31]. In CKD, plasma glutathione peroxidase activity is reduced, which mainly occurs due to deficient synthesis in damaged proximal tubule cells, which are the main source of plasma glutathione peroxidase [25]. Additionally, the immunosuppression in CKD gives rise to more frequent bacterial and viral infections, which induce more phagocytic activity and an increased release of ROS [32]. Studies have found that the influenza A M2 protein can interact with the mitochondrial antiviral signaling proteins on mitochondria to induce ROS production [33,34]. The overwhelming production of ROS stimulated by invading pathogens may induce DNA damage and mutation, inhibition of apoptosis, or activation of proto-oncogenes by initiating signal transduction pathways [35]. Oxidative stress can interact with all three cancer-development processes: initiation, promotion, and progression. ROS or reactive nitrogen species generate DNA lesions during initiation, contribute to abnormal gene expression (which results in increased cell proliferation during promotion), and generate a tumor microenvironment favorable for cancer progression [36].

In this study, we observed that vaccination had a dose-dependent effect, with adjusted HRs for lung cancer of 0.91, 0.49, and 0.25 when patients received 1, 2 or 3, and more than 4 vaccinations, respectively, during the follow-up period. Weng et al. demonstrated a similar phenomenon in a cohort study in which cumulative episodes of influenza were revealed to increase the risk of lung cancer, with adjusted ORs of 1.05 (95% CI 1.00–1.11), 1.12 (95% CI 1.00–1.25), and 1.25 (95% CI 1.13–1.39) in patients with 1–2, 3–4, and more than 5 episodes of influenza infection, respectively [17]. The results of the current study provide evidence for a potential link between inflammation attributed to influenza infection and lung tumor carcinogenesis, as has been suggested in other studies involving oncogenic viruses. The dose-dependent effect found in the current study strengthens the “hit-and-run,” “two hits,” and “multiple hits” theories with respect to viral oncogenesis [37,38]. However, no significant differences were observed after single-dose vaccination in the main model or in the subgroups, which supports the premise of a relationship between the accumulation of proinflammatory cytokines after influenza virus infection and lung cancer development. The results further emphasize the importance of annual influenza vaccination for patients with CKD.

Whether vaccinated or not, men had a higher incidence rate of lung cancer compared with women in our study. A similar effect was reported in a previous population-based cohort study in Australia, which revealed men, but not women, with stage 3 or higher stage of CKD had a significantly higher risk of cancer overall (test of interaction for sex; *p* = 0.004). The study also demonstrated the risk of lung and urinary tract cancers was higher among men with CKD [6]. A systematic review and other studies have revealed that a higher incidence rate of lung cancer among men in the worldwide general population may be attributed to a higher smoking rate in men, which is the leading risk factor of lung cancer [39,40].

This study had several limitations. First, several confounding factors (including smoke status, alcohol consumption status, and body mass index), and laboratory data such as eGFR could not be obtained from the NHIRD. However, we used propensity score method, which was applied for bias reduction in the comparison of a treatment to a non-randomized control group, to adjust our result [23,41]. Second, diagnosis of CKD and lung cancer and influenza vaccination status in our study were based on ICD-9-CM codes or drug codes; thus, the diagnoses used in our study relied on the diagnostic accuracy of the database. In the present study, we analyzed CKD patients who received hemodialysis. These patients represent more advance chronic disease. Third, various risk factors may lead to different histological types of lung cancer. Classifying lung cancer types according to histological type, such as small-cell lung cancer or non-small-cell lung cancer, may provide us with more robust data. Finally, we presumed that the patients took all prescribed medications with anti-cancer or anti-comorbidity associations, such as statins, metformin, aspirin, and drugs for the RAA system; this was to mitigate the effects of noncompliance but may have influenced our results. For the aforementioned problems to be resolved, further prospective clinical trials must be initiated, and more specific histological classifications of lung cancer must be made.

## 6. Conclusions

In conclusion, this study is the first to demonstrate the protective effects of influenza vaccination against lung cancer in patients with CKD. Moreover, this study demonstrated a dose-dependent effect on the reduction of the incidence of lung cancer in patients with CKD. In the future, large prospective clinical trials must be conducted to elucidate the underlying mechanisms proposed in this study.

## Figures and Tables

**Table 1:** Characteristics of sample population.

	Entire Cohort (n = 12,985)		Unvaccinated (n = 7490)		Vaccinated (n = 5495)		*p* ^a^
	n	%	n	%	n	%	
Age, years (mean ± SD)	70.98 (9.40)		70.09 (10.26)		72.18 (7.90)		<0.001
55–64	3989	30.72	2877	38.41	1112	20.24	<0.001
65–74	4541	34.97	2139	28.56	2402	43.71	
≥75	4455	34.31	2474	33.03	1981	36.05	
Sex							
Female	5712	43.99	3333	44.50	2379	43.29	0.172
Male	7273	56.01	4157	55.50	3116	56.71	
CCI^+^							
0	1491	11.48	876	11.70	615	11.19	0.013
1	2043	15.73	1166	15.57	877	15.96	
2	2876	22.15	1589	21.21	1287	23.42	
≥3	6575	50.64	3859	51.52	2716	49.43	
Dialysis							
No	9115	70.20	5371	71.71	3744	68.13	<0.001
Yes	3870	29.80	2119	28.29	1751	31.87	
COPD							
No	8224	63.33	4746	63.36	3478	63.29	0.934
Yes	4761	36.67	2744	36.64	2017	36.71	
Lower respiratory tract infection							
No	10,649	82.01	5952	79.47	4697	85.48	<0.001
Yes	2336	17.99	1538	20.53	798	14.52	
Influenza							
No	9593	73.88	5411	72.24	4182	76.11	<0.001
Yes	3392	26.12	2079	27.76	1313	23.89	
Diabetes							
No	6310	48.59	3355	44.79	2955	53.78	<0.001
Yes	6675	51.41	4135	55.21	2540	46.22	
Hypertension							
No	2555	19.68	1387	18.52	1168	21.26	<0.001
Yes	10,430	80.32	6103	81.48	4327	78.74	
Dyslipidemia							
No	6337	48.80	3386	45.21	2951	53.70	<0.001
Yes	6648	51.20	4104	54.79	2544	46.30	
Statin							
<28 days	7972	61.39	4786	63.90	3186	57.98	<0.001
28–365 days	2683	20.66	1576	21.04	1107	20.15	
>365 days	2330	17.94	1128	15.06	1202	21.87	
Metformin							
<28 days	10,266	79.06	6045	80.71	4221	76.82	<0.001
28–365 days	1331	10.25	804	10.73	527	9.59	
>365 days	1388	10.69	641	8.56	747	13.59	
RAA							
<28 days	4114	31.68	2792	37.28	1322	24.06	<0.001
28–365 days	3751	28.89	2344	31.30	1407	25.61	
>365 days	5120	39.43	2354	31.43	2766	50.34	
Aspirin							
<28 days	6715	51.71	4478	59.79	2237	40.71	<0.001
28–365 days	3149	24.25	1702	22.72	1447	26.33	
>365 days	3121	24.04	1310	17.49	1811	32.96	
Level of urbanization							
Urban	8785	67.65	5350	71.43	3435	62.51	<0.001
Suburban	2806	21.61	1488	19.87	1318	23.99	
Rural	1394	10.74	652	8.70	742	13.50	
Monthly income (NT$)							
0	1596	12.29	901	12.03	695	12.65	<0.001
1–21,000	4486	34.55	2397	32.00	2089	38.02	
21,000–33,300	3788	29.17	1996	26.65	1792	32.61	
≥33,301	3115	23.99	2196	29.32	919	16.72	

^a^ Comparison between unvaccinated and vaccinated, CCI^+^: Charlson comorbidity index.

**Table 2:** Risk of lung cancer among unvaccinated and vaccinated groups in the study cohort.

All Groups (n = 12,985)	Unvaccinated (Total Follow-Up: 21,919.2 Person-Years; Mean Period of Follow-Up: 2.93 Person-Years)				Vaccinated (Total Follow-Up: 33,990.2 Person-Years; Mean Period of Follow-Up: 6.19 Person-Years)				Adjusted HR ^†^ (95% CI)
	No. of Patients with Cancer	Incidence Rate (Per 10^5^ Person-Years) (95% CI)			No. of Patients with Cancer	Incidence Rate (Per 10^5^ Person-Years) (95% CI)			
Entire cohort	140	638.7	(532.9,	744.5)	119	350.1	(287.2,	413.0)	0.50 (0.38, 0.65) ***
Stratified by age									
Age, 55–64 ^a^	46	426.4	(303.2,	549.6)	15	166.7	(82.3,	251.1)	0.39 (0.21, 0.70) **
Age, 65–74 ^b^	40	652.1	(450.0,	854.2)	55	347.6	(255.7,	439.5)	0.50 (0.33, 0.76) ***
Age, ≥75 ^c^	54	1080.5	(792.3,	1368.7)	49	534.3	(384.7,	683.9)	0.49 (0.32, 0.72) ***
Stratified by sex									
Female ^d^	44	456.6	(321.7,	591.5)	27	180.2	(112.3,	248.2)	0.39 (0.24, 0.65) ***
Male ^e^	96	781.6	(625.2,	937.9)	92	483.9	(385.1,	582.8)	0.55 (0.40, 0.74) ***
Stratified by Dialysis									
No ^f^	98	660.7	(529.9,	791.5)	81	356.6	(278.9,	434.2)	0.47 (0.34, 0.64) ***
Yes ^g^	42	592.7	(413.5,	772.0 )	38	337.1	(229.9,	444.3)	0.55 (0.35, 0.88) *
Stratified by COPD									
No ^h^	65	416.6	(315.3,	517.9)	64	272.3	(205.6,	339.0)	0.52 (0.36, 0.76) ***
Yes ^i^	75	1187.4	(918.7,	1456.1)	55	524.4	(385.8,	662.9)	0.44 (0.30, 0.64) ***
Stratified by Lower respiratory tract infection									
No ^j^	99	517.7	(415.7,	619.6)	91	297.3	(236.2,	358.4)	0.47 (0.34, 0.63) ***
Yes ^k^	41	1467.0	(1018.0,	1916.1)	28	828.6	(521.7,	1135.5)	0.51 (0.31, 0.85) **
Stratified by Influenza									
No ^l^	98	580.6	(465.7,	695.6)	90	330.6	(262.3,	398.9)	0.50 (0.37, 0.67) ***
Yes ^m^	42	833.2	(581.2,	1085.1)	29	428.5	(272.5,	584.4)	0.50 (0.30, 0.82) **

* *p* < 0.05; ** *p* < 0.01; *** *p* < 0.001. ^a^. Total follow-up: 10,787.5 person-years for unvaccinated and 8998.0 for vaccinated. ^b^. Total follow-up: 6134.2 person-years for unvaccinated and 15,821.9 for vaccinated. ^c^. Total follow-up: 4997.5 person-years for unvaccinated and 9170.3 for vaccinated. ^d^. Total follow-up: 9636.6 person-years for unvaccinated and 14,979.9 for vaccinated. ^e^. Total follow-up: 12,282.6 person-years for unvaccinated and 19,010.3 for vaccinated. ^f^. Total follow-up: 14,833.5 person-years for unvaccinated and 22,716.8 for vaccinated. ^g^. Total follow-up: 7085.7 person-years for unvaccinated and 11,273.4 for vaccinated. ^h^. Total follow-up: 15,602.9 person-years for unvaccinated and 23,501.3 for vaccinated. ^i^. Total follow-up: 6316.3 person-years for unvaccinated and 10,489.0 for vaccinated. ^j^. Total follow-up: 19,124.5 person-years for unvaccinated and 30,611.0 for vaccinated. ^k^. Total follow-up: 2794.8 person-years for unvaccinated and 3379.2 for vaccinated. ^l^. Total follow-up: 16,878.2 person-years for unvaccinated and 27,222.1 for vaccinated. ^m^. Total follow-up: 5041.1 person-years for unvaccinated and 6768.2 for vaccinated. CI: confidence interval. HR: hazard ratio. ^†^ Main model propensity score adjusted for age, sex, Charlson comorbidity index, dialysis, COPD, lower respiratory tract infection, influenza, diabetes, hypertension, dyslipidemia, level of urbanization, and monthly income.

**Table 3:** Sensitivity Analysis of Adjusted HRs of Vaccination for Risk Reduction in Lung Cancer.

	Unvaccinated	Vaccinated			*p* for Trend
		1	2–3	≥4	
	Adjusted HR (95% CI)	Adjusted HR (95% CI)	Adjusted HR (95% CI)	Adjusted HR (95% CI)	
Main model ^†^	1.00	0.91 (0.66, 1.25)	0.49 (0.34, 0.71) ***	0.25 (0.17, 0.38) ***	<0.001
Additional covariates ^‡^					
Main model + statin	1.00	0.93 (0.67, 1.29)	0.52 (0.36, 0.74) ***	0.27 (0.18, 0.41) ***	<0.001
Main model + metformin	1.00	0.93 (0.67, 1.28)	0.51 (0.35, 0.73) ***	0.26 (0.17, 0.40) ***	<0.001
Main model + RAA	1.00	0.96 (0.70, 1.33)	0.52 (0.36, 0.75) ***	0.28 (0.18, 0.42) ***	<0.001
Main model + aspirin	1.00	0.95 (0.69, 1.31)	0.52 (0.36, 0.75) ***	0.28 (0.18, 0.42) ***	<0.001
Subgroup effects					
Age, years					
55–64	1.00	0.56 (0.25, 1.24)	0.49 (0.21, 1.15)	0.14 (0.03, 0.58) **	<0.001
65–74	1.00	1.22 (0.74, 2.03)	0.51 (0.29, 0.91) *	0.22 (0.12, 0.41) ***	<0.001
≥75	1.00	0.77 (0.46, 1.28)	0.42 (0.24, 0.73) **	0.33 (0.17, 0.62) ***	<0.001
Sex					
Female	1.00	0.68 (0.36, 1.30)	0.47 (0.24, 0.93) *	0.13 (0.04, 0.37) ***	<0.001
Male	1.00	1.03 (0.71, 1.50)	0.51 (0.33, 0.78) ***	0.30 (0.19, 0.48) ***	<0.001
CCI^+^					
0	1.00	3.33 (1.16, 9.55) *	1.06 (0.31, 3.66)	0.89 (0.29, 2.78)	0.602
1	1.00	0.48 (0.20, 1.16)	0.40 (0.16, 0.98) *	0.22 (0.09, 0.55) ***	0.001
2	1.00	1.00 (0.51, 1.95)	0.79 (0.41, 1.51)	0.23 (0.10, 0.54) ***	0.001
≥3	1.00	0.81 (0.52, 1.28)	0.33 (0.19, 0.59) ***	0.20 (0.10, 0.39) ***	<0.001
Dialysis					
No	1.00	0.93 (0.63, 1.36)	0.42 (0.26, 0.66) ***	0.24 (0.15, 0.39) ***	<0.001
Yes	1.00	0.84 (0.46, 1.54)	0.65 (0.36, 1.17)	0.27 (0.12, 0.58) ***	<0.001
COPD					
No	1.00	1.01 (0.64, 1.59)	0.55 (0.33, 0.90) *	0.25 (0.14, 0.45) ***	<0.001
Yes	1.00	0.76 (0.48, 1.21)	0.40 (0.24, 0.68) ***	0.24 (0.13, 0.44) ***	<0.001
Lower respiratory tract infection					
No	1.00	0.90 (0.62, 1.31)	0.49 (0.32, 0.74) ***	0.22 (0.14, 0.36) ***	<0.001
Yes	1.00	0.79 (0.42, 1.48)	0.42 (0.19, 0.91) *	0.32 (0.14, 0.75) **	0.002
Influenza infection					
No	1.00	0.95 (0.65, 1.39)	0.47 (0.30, 0.72) ***	0.27 (0.17, 0.42) ***	<0.001
Yes	1.00	0.77 (0.41, 1.45)	0.57 (0.29, 1.14)	0.20 (0.08, 0.52) ***	<0.001
Diabetes					
No	1.00	0.90 (0.59, 1.38)	0.49 (0.31, 0.78) **	0.26 (0.16, 0.43) ***	<0.001
Yes	1.00	0.93 (0.57, 1.54)	0.51 (0.28, 0.91) *	0.21 (0.09, 0.50) ***	<0.001
Dyslipidemia					
No	1.00	0.91 (0.59, 1.39)	0.51 (0.32, 0.80) **	0.27 (0.16, 0.44) ***	<0.001
Yes	1.00	0.94 (0.57, 1.56)	0.49 (0.27, 0.88) *	0.22 (0.10, 0.51) ***	<0.001
Hypertension					
No	1.00	1.13 (0.61, 2.08)	0.66 (0.34, 1.27)	0.15 (0.06, 0.38) ***	<0.001
Yes	1.00	0.84 (0.58, 1.23)	0.44 (0.28, 0.68) ***	0.30 (0.19, 0.48) ***	<0.001
Statin					
<28 days	1.00	1.01 (0.70, 1.44)	0.51 (0.34, 0.78) **	0.23 (0.13, 0.38) ***	<0.001
28–365 days	1.00	1.00 (0.42, 2.41)	0.68 (0.28, 1.68)	0.24 (0.07, 0.87) *	0.031
>365 days	1.00	0.34 (0.08, 1.50)	0.35 (0.10, 1.27)	0.52 (0.20, 1.34)	0.146
Metformin					
<28 days	1.00	0.82 (0.57, 1.18)	0.53 (0.36, 0.78) ***	0.23 (0.14, 0.37) ***	<0.001
28–365 days	1.00	1.59 (0.55, 4.61)	0.50 (0.11, 2.43)	0.86 (0.21, 3.58)	0.583
>365 days	1.00	1.91 (0.64, 5.69)	0.20 (0.02, 1.65)	0.37 (0.09, 1.51)	0.064
RAA					
<28 days	1.00	0.97 (0.58, 1.63)	0.37 (0.18, 0.76) **	0.13 (0.05, 0.36) ***	<0.001
28–365 days	1.00	0.98 (0.56, 1.71)	0.58 (0.32, 1.04)	0.20 (0.08, 0.48) ***	<0.001
>365 days	1.00	1.09 (0.58, 2.04)	0.72 (0.38, 1.36)	0.50 (0.27, 0.93) *	0.018
Aspirin					
<28 days	1.00	0.85 (0.54, 1.34)	0.61 (0.37, 0.99) *	0.16 (0.07, 0.36) ***	<0.001
28–365 days	1.00	1.26 (0.71, 2.26)	0.37 (0.17, 0.81) *	0.38 (0.19, 0.79) **	0.001
>365 days	1.00	0.86 (0.39, 1.90)	0.59 (0.27, 1.29)	0.35 (0.16, 0.77) **	0.006

* *p* < 0.05; ** *p* < 0.01; *** *p* < 0.001. HR: hazard ratio. CCI^+^: Charlson comorbidity index. ^†^ Main model propensity score adjusted for age, sex, Charlson comorbidity index, dialysis, COPD, lower respiratory tract infection, influenza infection, diabetes, hypertension, dyslipidemia, level of urbanization, and monthly income. ^‡^ Models were adjusted for covariates in the main model and for each additionally listed covariate.

## Data Availability

The data supporting the findings of the present research were sourced from the NHIRD in Taiwan. Due to legal restrictions imposed by the government of Taiwan related to the Personal Information Protection Act, the database cannot be made publicly available. However, upon reasonable request of the authors and with permission from the NHIRD, the relevant data are available.
